# Exploring the determinants of fruits and vegetables consumption among adults in Saudi Arabia: integrating habit strength in theory of planned behavior

**DOI:** 10.3389/fnut.2025.1568912

**Published:** 2025-05-09

**Authors:** Buthaina M. Aljehany, Haya Aljadani, Howeida Abusalih, Eman A. Abduljawad

**Affiliations:** ^1^Food and Nutrition Department, Human Sciences and Design Faculty, King Abdulaziz University, Jeddah, Saudi Arabia; ^2^Department of Health Sciences, College of Health and Rehabilitation Sciences, Princess Nourah Bint Abdulrahman University, Riyadh, Saudi Arabia

**Keywords:** theory of planned behavior, intention, habits, moderator, structural equation modeling, fruits and vegetables consumption, Saudi Arabia

## Abstract

**Introduction:**

Fruit and vegetable consumption is low among adults in Saudi Arabia.

**Aim:**

To identify the main determinants of fruit and vegetable consumption among Saudi adults and to suggest possible intervention strategies to promote healthier eating habits.

**Methods:**

A cross-sectional study was conducted with a sample of Saudi adult students (*n* = 471), using the Theory of Planned Behavior (TPB) as a conceptual framework. Structural equation modeling (SEM) was used to test the TPB predictors for consuming the recommended five servings of fruits and vegetables per day in the upcoming week, and multi-group analysis was conducted to assess the moderating effect of habits.

**Results:**

The findings indicate that both the average daily servings and the frequency of meeting the recommended five servings per day are notably low. Higher fruit and vegetable consumption habits are associated with better knowledge, healthier dietary practices, and more consistent physical activity. Perceived Behavioral Control (PBC) emerged as a significant predictor of fruit and vegetable consumption behavior among individuals with low consumption habits.

**Conclusion:**

Fruit and vegetable consumption is influenced by both intention and PBC, with individual habits being an important consideration. To increase fruits and vegetables intake, interventions should be tailored based on the individual’s level of habit.

## 1 Introduction

Fruits and vegetables are fundamental to a healthy, balanced diet, providing a broad spectrum of health benefits, including a reduced risk of chronic diseases, such as hypertension, coronary heart disease, and stroke (1), prevention of weight gain and retardation of the onset or progression of other geriatric conditions (2). Reflecting this scientific evidence, the World Health Organization (WHO) and the Food and Agriculture Organization (FAO) recommend a minimum intake of 400 grams of fruits and vegetables per person per day (excluding potatoes and other starchy tubers) (3). This recommendation is commonly promoted through public health initiatives, such as the “Five-a-Day” guideline, which encourages individuals to consume at least five servings of fruits and vegetables daily (4). However, despite these well-established recommendations, global adherence to these guidelines remains suboptimal, highlighting the need for continued efforts to enhance fruit and vegetable consumption worldwide. To address the issue of insufficient fruit and vegetable consumption, researchers are increasingly drawing on theories from psychology, health, and sociology. These theories help them understand the factors that influence our food choices and design interventions to promote healthier eating habits. One such prominent theory is the Theory of Planned Behavior (TPB), a well-established psychological model used to predict and understand health-related behaviors (5). A comprehensive review by Yan (6) emphasized the utility of the TPB in analyzing dietary behaviors and providing insights for intervention design. Research by Sjoberg et al. (7) demonstrated that key TPB constructs—such as perceived behavioral control, attitudes, and subjective norms—accounted for a significant proportion of the variance in both the intention to consume fruits and vegetables and actual intake among older adults. Similarly, Kothe et al. (8) evaluated a TPB-based intervention and observed significant increases in fruit and vegetable consumption, along with positive changes in the TPB-related variables. Further supporting the framework’s relevance, Solhi and Shirzad (9) found that intention, perceived behavioral control, and enabling factors were strong predictors of fruit and vegetable consumption among female dormitory students. These studies collectively highlight the TPB’s applicability in understanding and promoting healthier eating habits across various populations, making it a valuable tool for shaping dietary behaviors and improving fruit and vegetable intake (10).

A recent review highlighted the widespread issue of inadequate fruit and vegetable consumption, particularly in Middle Eastern and North African countries, with Saudi Arabia being a notable concern. A national health survey conducted in 2013 revealed that 97.4% of Saudi adults consumed fewer than five servings of fruits and vegetables per day, and only 2.6% met the recommended intake. More recent data from Albrahim et al. (11) found that 91.6% of participants in Riyadh consumed fewer than two servings daily. Therefore, several studies have explored factors contributing to low fruit and vegetable consumption in Saudi Arabia. These include determinants such as fitness consciousness, physical activity, living with family, and meal planning (12), self-efficacy (13) and perceived benefits and barriers to healthy eating (14). Despite the Saudi Ministry of Health’s (15) recommendation of five servings of fruits and vegetables per day, and numerous studies underscoring the importance of addressing this dietary behavior, none have specifically investigated its determinants using the Theory of Planned Behavior (TPB). This gap in research underscores the need for further investigation into the factors influencing fruit and vegetable consumption in Saudi Arabia. Therefore, the objective of this study is to provide an in-depth analysis of fruit and vegetable consumption among Saudi adults through the application of the TPB.

## 2 Theoretical framework

The theoretical framework for this study is based on the model developed by Menozzi et al. (16), which employs the Theory of Planned Behavior (TPB) to predict health-related intentions and behaviors. The TPB has been widely applied across various fields, including health behaviors (17, 18), and more specifically in understanding fruit and vegetable consumption (19). This model posits that individual behavior is influenced by three primary factors: attitude, subjective norms (SN), and perceived behavioral control (PBC), all of which contribute to the formation of behavioral intentions that, in turn, predict actual behavior. This study, grounded in the Theory of Planned Behavior (TPB), proposes several hypotheses to explore the factors influencing fruit and vegetable consumption among Saudi adults. It is hypothesized that a positive attitude will significantly predict the intention to consume fruits and vegetables. Additionally, it is expected that subjective norms will play a significant role in shaping these intentions. Perceived behavioral control (PBC) is also anticipated to significantly influence both the intention to consume fruits and vegetables and the actual behavior. Furthermore, the study proposes that intention will be a strong predictor of actual fruit and vegetable consumption. Finally, it is hypothesized that PBC will have a direct effect on actual consumption, further influencing dietary behavior. These hypotheses aim to identify the key factors that contribute to fruit and vegetable consumption in Saudi Arabia, providing insights for targeted interventions.

While the Theory of Planned Behavior (TPB) has been widely applied to predict intentions and behaviors in various fields, including health (17, 20, 21), it may not fully capture the complexity of behaviors such as food choices (22). Research has shown that habit plays a significant role in food-related behaviors, including fruit and vegetable consumption (16, 23, 24). In particular, habit has been found to moderate the relationship between intention and behavior, as seen in studies of fruit consumption (25). This paper aims to (1) validate the TPB predictors of fruits and vegetable consumption among Saudi adults and (2) examine the role of habit in moderating the intention-behavior relationship. Additionally, it will analyze relevant beliefs to inform the development of targeted interventions aimed at promoting healthier dietary behaviors.

## 3 Methods

### 3.1 Study population and study design

This cross-sectional study was conducted across Saudi Arabia from May to October 2024. Inclusion criteria in the study were being Saudi citizens, residing in Saudi Arabia, and aged ≥18 years old. Exclusion criteria will include pregnant or lactating women, individuals on special diets, or those with chronic diseases that may significantly influence their fruit and vegetable intake. Ethical approval for the study was granted by the Institutional Review Board (IRB) of King Abdulaziz University (IRB Log number 15–24). Informed consent was obtained from all participants prior to their participation, with consent provided on the first page of the questionnaire. Participants were fully informed about the purpose of the study, the voluntary nature of their involvement, and the specific requirements of their participation. They were also notified that they could withdraw from the study at any time without consequence while completing the questionnaire. To ensure participant confidentiality, no personally identifiable information was collected, and all data were securely stored. The data collected were used exclusively for research purposes and remained confidential throughout the study.

### 3.2 Sampling technique and sample size calculation

Recruitment for this study was conducted via an online, self-administered questionnaire hosted on Google Forms. The electronic questionnaire was distributed through social media platforms such as WhatsApp and Telegram. Given the nature of the distribution, a non-probability, convenience sampling technique was employed. The sample size was determined using a non-probability sampling approach, with an assumed prevalence rate of 50% due to the lack of national data on the proportion of Saudi adults consuming five servings of fruits and vegetables per day. Based on the total population of Saudi adults, which is approximately 13 million according to The Saudi Census (26), the initial sample size was calculated to be 385 participants using the appropriate sample size formula. To account for potential incomplete data or non-responses, an additional 10% was added to the calculated sample size, resulting in a minimum final sample size of 422 participants. Hence, the total final sample was *n* = 471.

### 3.3 Data collection tool

The study used a structured questionnaire consisting of the following sections:

*Section 1 – General Information*: This section collected demographic and background data, including participants’ age, gender, marital status, education level, region of residence, monthly income (SAR).
*Section 2 – Nutrition Knowledge*: This section composed of two questions to assess participants’ knowledge about nutrition, focusing on their awareness of nutrition sources and their understanding of the recommended daily servings of fruits and vegetables, in line with the guidelines from the Saudi Ministry of Health (15).
*Section 3 – Overall Lifestyle Behaviors*: This section included several questions designed to examine participants’ overall lifestyle behaviors. Topics covered physical activity, meal-sharing habits with family, dietary choices, and responsibilities related to purchasing and preparing fruits and vegetables.
*Section 4: The TPB constructs items*: The study was based on Ajzen’s conceptual framework for developing a Theory of Planned Behavior (TPB) questionnaire (5, 27). The Target, Action, Context, and Time (TACT) strategy was employed to define the behavior being assessed, which was “eating 5 servings of fruits and vegetables per day next week.” The TACT elements were determined in line with the recommendations of the Saudi Arabian Ministry of Health. Consistent with the World Health Organization’s (WHO) definition, potatoes, sweet potatoes, and other starchy roots were excluded from the category of vegetables.
*Attitude*: It will be measured with 4 semantic differentials (e.g., “Eating at least 5 servings of vegetables and fruits per day next week is bad/good, enjoyable/not enjoyable, beneficial/unbeneficial and easy to digest/difficult to digest”).
*Subjective norms*: It will be assessed by 2 items as follows: “My family/friends expect me to eat 5 servings of fruits and vegetables per day next week.”
*Normative belief*: For this component, 2 items will be used to measure this component related to a particular referent individual of group (family and friends) thinks respondents should or should not perform the behavior of eating 5 servings of fruits and vegetables a day.
*Behavioral beliefs*: It is focuses on the belief in outcomes of behavior, 4 items will be used which are: If I consumed to eat 5 servings of fruits and vegetables a day, in my opinion, I would have more energy/be feeling better/be having higher control over weigh/be healthier in the future.”
*Control belief*: It is about factors that would enable or impede, behavior performance will measured assessed considering 6 items: “Under control,” High cost,” “Low cooking skills,” “Conservation difficulty,” “Pesticide residuals”; and “Not promoted by companies.”
*Perceived behavioral control (PBC)*: Two items will be employed to measure the PCB which are: “I think that eating to eat 5 servings of fruits and vegetables a day next week is something I can achieve” and “I am confident that I can eat 5 servings of fruits and vegetables a day next week.”
*Behavioral intention*: will be evaluated by 3 items which are: “I intend/I am sure/My goal is to eat 5 servings of fruits and vegetables per day next week.”
*Behavior*: the consumption of 5 servings of fruits and vegetables per day. This frequency will be assessed by 2 questions which are: How many servings of fruits and vegetables have you eaten per day in the last week? And “How many times have you eaten 5 servings of fruits and vegetables per day in the last week?”
*Section 5*: Consisting in the Self-Report Habit Index. It is composed of 4 items considering the different aspect of the habit based on the short version of the (28, 29): “*Consuming at least 5 serving of fruits and vegetables a day is something that: I do automatically/that makes me feel weird if I do not do it/that belongs to my daily routine/that makes me feel well if I do it*.”

Each of the items will be scored on 5 points Likert scale ranging from 1 to 5, (in general, 1 = Strongly disagree, 2- disagree, 3- Neutral, 4- Agree, and 5- Strongly agree). For each of the TPB constructs a total scoring will be calculated and computed for each participant.

### 3.4 Validity

Once the questionnaire items were finalized, the instrument was distributed to a panel of 11 experts in nutrition, public health, and psychology for evaluation of its face validity (30). The evaluation focused on three key aspects as outlined by Ehrenbrusthoff et al. (31): content completeness, clarity, and the time required to complete the questionnaire. The results indicated that the content was deemed comprehensive by 88.9, easily understood by 88.9, and the time required to complete the questionnaire averaged 8.9 ± 1.4. Experts were also asked to provide feedback on the questions to assess the overall usability of the instrument, identifying any potential content or linguistic ambiguities that might require revision, as recommended by Janssens et al. (32). Based on their feedback, necessary revisions were implemented to ensure the questionnaire is both valid and user-friendly.

In addition to face validity, content validity was also assessed. The Content Validity Index (CVI) for individual items (I-CVI) ranged from 0.82 to 1, with an expert agreement proportion of 97.1%. The Content Validity Index for the scale (S-CVI)/Average was 93.3%, and the Content Validity Index for the scale (S-CVI)/Universal Agreements was 77.4%. These results confirm the high validity of the questionnaire in terms of content.

### 3.5 Statistical analysis

All data analyses were conducted using the R software. The items of the TPB were assessed using a 5-point Likert scale. Attitudes toward the behavior were measured with four differential items. Subjective norms were evaluated using two items, and PBC was assessed with two items as well. Behavioral intention was measured using three items, while two items were used to evaluate the actual behavior. Habit was measured with four items representing different aspects of habitual behavior. The participants were grouped into three habit categories based on tertiles of the mean index score, resulting in nearly equal group sizes: low habit (<2.5, *n* = 153), medium habit (2.6–3.24, *n* = 152), and high habit (≥3.25, *n* = 167) (Table 1). The Cronbach’s alpha values for all constructs indicate good internal consistency, with values ranging from 0.72 to 0.92. The internal consistency of the scales, as indicated by Cronbach’s alpha, suggested that the scales were relatively homogeneous. The data were initially analyzed to identify major differences in TPB variables across habit groups and to confirm the relationships between attitude, subjective norms, and PBC with their respective behavioral, normative, and control beliefs, as well as between these predictors and both intention and behavior. A structural equation modeling (SEM) approach was employed to test the research hypotheses. Model fit was assessed using several indices, including chi-square (c2), comparative fit index (CFI), Tucker-Lewis index (TLI), root mean square error of approximation (RMSEA), and standardized root mean square residual (SRMR), with R^2^ used to measure the explained variance of the endogenous variables (intention and behavior). A good model fit was considered when CFI and TLI were greater than 0.90, and RMSEA and SRMR were less than 0.08. The models were estimated using the Maximum Likelihood estimator.

**Table 1:** General information of the studied population according to the habit level (*n* = 471).

Studied parameters	Total *N* = 471	Low habit *N* = 153	Medium habit *N* = 151	High habit *N* = 167	*p*-value
Age	34.53 ± 13.86	31.08 ± 12.43	34.49 ± 13.32	37.72 ± 14.88	<0.001*
Gender					
Female	352.00 (74.58%)	105.00 (68.63%)	114.00 (75.00%)	133.00 (79.64%)	0.090
Male	113.00 (23.94%)	45.00 (29.41)	36.00 (23.68%)	32.00 (19.16%)	
BMI					
<18.5 kg/m2	34.00 (7.61%)	18.00 (12.24%)	8.00 (5.48%)	8.00 (5.10%)	0.094
18.5–24.9	196.00 (43.56%)	57.00 (38.78%)	66.00 (45.21%)	73.00 (46.50%)	
25–29.9	116.00 (25.95%)	33.00 (22.45%)	37.00 (25.34%)	46.00 (29.30%)	
>30	104.00 (23.11%)	39.00 (26.53%)	35.00 (23.97%)	30.00 (19.11%)	
Marital status					
Married	215.00 (45.55%)	57.00 (37.25%)	72.00 (47.37%)	86.00 (51.50%)	0.019*
Not married	251.00 (53.18%)	96.00 (62.75%)	78.00 (51.32%)	77.00 (46.11%)	
Educational level					
Secondary/higher	44.00 (9.32%)	11.00 (7.19%)	16.00 (10.53%)	17.00 (10.18%)	0.200
Bachelor/diploma	239.00 (50.64%)	86.00 (56.21%)	65.00 (42.76%)	88.00 (52.69%)	
Postgraduate	181.00 (38.35%)	54.00 (35.29%)	67.00 (44.08%)	60.00 (35.93%)	
Place origin					
Northern	11.00 (2.33%)	4.00 (2.61%)	2.00 (1.32%)	5.00 (2.99%)	0.603
Southern	22.00 (4.66%)	7.00 (4.58%)	10.00 (6.58%)	5.00 (2.99%)	
Middle	129.00 (27.33%)	35.00 (22.88%)	45.00 (29.61%)	49.00 (29.34%)	
Eastern	7.00 (1.48%)	2.00 (1.31%)	2.00 (1.32%)	3.00 (1.80%)	
Western	295.00 (62.50%)	105.00 (68.63%)	89.00 (58.55%)	101.00 (60.48%)	
Monthly income					
<5,000	53.00 (11.23%)	21.00 (13.73%)	18.00 (11.84%)	14.00 (8.38%)	0.552
5,000–10,000	134.00 (28.39%)	42.00 (27.45%)	48.00 (31.58%)	44.00 (26.35%)	
11,000–20,000	162.00 (34.32%)	54.00 (35.29%)	48.00 (31.58%)	60.00 (35.93%)	
>20,000	115.00 (24.36%)	35.00 (22.88%)	33.00 (21.71%)	47.00 (28.14%)	

Bold value means a significant *p*-value.

To assess the moderating role of habit in the relationship between PBC, intentions, and behavior, multi-group SEM was employed to test for significant differences across the three habit groups. The validity of the baseline model (configural model) was first tested separately for each group, followed by a simultaneous test for cross-group equivalence. Measurement invariance was then evaluated to determine whether each item was equivalent across the groups. Finally, after establishing metric and scalar equivalence, the invariance of the structural model was tested by examining factor covariances and structural paths. The invariance was assessed using several fit indices, including the *χ*^2^ difference (Δ*χ*^2^) test, CFI difference (ΔCFI), TLI difference (ΔTLI), RMSEA difference (ΔRMSEA), and SRMR difference (ΔSRMR). Evidence of invariance was considered valid if the Δ*χ*^2^ was non-significant, ΔCFI and ΔTLI were both less than 0.01, ΔRMSEA was below 0.015, and ΔSRMR was under 0.02.

## 4 Results

### 4.1 Descriptive analysis

Table 1 presents several demographic, health, and lifestyle characteristics of participants across three fruits and vegetables consumption habit groups: low, medium, and high. The results highlight significant differences in various variables, as well as trends across these groups. There was a significant difference in age across the three groups (*p* < 0.001). Participants in the high habit group were significantly older (37.72 ± 14.88 years) compared to those in the low habit group (31.08 ± 12.43 years), A significant difference in marital status was found (*p* = 0.019). A higher percentage of participants in the low habit group were unmarried (62.75%) compared to the high habit group (46.11%), which had a higher proportion of married individuals. The gender, Region, Monthly income, and BMI, Educational level distribution showed no significant differences across the groups, showing similar distribution across the groups with a higher proportion of females in all groups, though this was consistent across low, medium, and high habit groups.

Table 2 presents the general characteristics of the studied population based on their knowledge of dietary source and consumption recommendations as well as fruit and vegetable consumption habits, categorized into low, medium, and high habit levels. The study reveals significant differences across these groups, particularly in areas such as family meal frequency, fruit and vegetable purchasing, and preparation habits, all showing strong associations with habit levels (*p*-values <0.001). There were no significant differences in sources of nutrition knowledge. Most participants across all groups obtained health information from social media (45.34% overall). In terms of family meal sharing, a significant difference was found with the high habit group share a family meal (56.29%), while the low habit group had a higher proportion of participants less frequently family meal (5.08%) reporting never sharing a family meal (*p* < 0.001). In the low habit group, a higher percentage (34.64%) was unaware of the correct recommendation, with only 9.15% reporting the correct knowledge of 5 serving. In contrast, the high habit group showed a greater proportion through the groups with 22.75% aware of the correct recommendation, suggesting that individuals with higher consumption habits have more accurate knowledge of dietary guidelines (*p*-value 0.025). Additionally, dietary intake and physical activity also showed notable differences between habit levels, with those in the high habit group reporting healthier dietary choices and more frequent physical activity. For instance, high-habit participants had the highest frequency of purchasing and preparing fruits and vegetables, in contrast to low-habit individuals.

**Table 2:** Knowledge, dietary practices and physical activity of the studied population according to the habit level (*n* = 471).

Studied parameters	Total *N* = 471	Low habit *N* = 153	Medium habit *N* = 151	High habit *N* = 167	*p*-value
Sources of nutrition knowledge					
Health	120.00 (25.42%)	32.00 (20.92%)	34.00 (22.37%)	54.00 (32.34%)	0.213
Relatives and friends	35.00 (7.42%)	12.00 (7.84%)	15.00 (9.87%)	8.00 (4.79%)	
Social media	214.00 (45.34%)	77.00 (50.33%)	66.00 (43.42%)	71.00 (42.51%)	
WHO	67.00 (14.19%)	21.00 (13.73%)	21.00 (13.82%)	25.00 (14.97%)	
Other	31.00 (6.57%)	10.00 (6.54%)	13.00 (8.55%)	8.00 (4.79%)	
Knowledge regarding the WHO dietary consumption recommendations					
<3	140.00 (29.66%)	53.00 (34.64%)	42.00 (27.63%)	45.00 (26.95%)	0.025*
3	183.00 (38.77%)	62.00 (40.52%)	59.00 (38.82%)	62.00 (37.13%)	
4	54.00 (11.44%)	18.00 (11.76%)	18.00 (11.84%)	18.00 (10.78%)	
5	72.00 (15.25%)	14.00 (9.15%)	20.00 (13.16%)	38.00 (22.75%)	
≥6	18.00 (3.81%)	5.00 (3.27%)	10.00 (6.58%)	3.00 (1.80%)	
Family meals					
Never	24.00 (5.08%)	17.00 (11.11%)	5.00 (3.29%)	2.00 (1.20%)	<0.001*
1–2 days/week	89.00 (18.86%)	33.00 (21.57%)	30.00 (19.74%)	26.00 (15.57%)	
3–4 days/week	76.00 (16.10%)	24.00 (15.69%)	25.00 (16.45%)	27.00 (16.17%)	
5–6 days/week	59.00 (12.50%)	24.00 (15.69%)	18.00 (11.84%)	17.00 (10.18%)	
Daily	218.00 (46.19%)	53.00 (34.64%)	71.00 (46.71%)	94.00 (56.29%)	
Fruits and vegetables purchasing					
Never	15.00 (3.18%)	12.00 (7.84%)	2.00 (1.32%)	1.00 (0.60%)	<0.001*
Rare	44.00 (9.32%)	21.00 (13.73%)	16.00 (10.53%)	7.00 (4.19%)	
Sometimes	114.00 (24.15%)	45.00 (29.41%)	40.00 (26.32%)	29.00 (17.37%)	
Often	140.00 (29.66%)	48.00 (31.37%)	50.00 (32.89%)	42.00 (25.15%)	
Always	154.00 (32.63%)	27.00 (17.65%)	41.00 (26.97%)	86.00 (51.50%)	
Fruits and vegetables cooking/preparing					
Never	52.00 (11.02%)	30.00 (19.61%)	11.00 (7.24%)	11.00 (6.59%)	<0.001*
Rare	62.00 (13.14%)	30.00 (19.61%) 35.00 (22.88%)	17.00 (11.18%)	15.00 (8.98%)	
Sometimes	105.00 (22.25%)	32.00 (20.92%)	46.00 (30.26%)	24.00 (14.37%)	
Often	119.00 (25.21%)	25.00 (16.34%)	40.00 (26.32%)	47.00 (28.14%)	
Always	127.00 (26.91%)	30.00 (19.61%)	33.00 (21.71%)	69.00 (41.32%)	
Dietary intake					
Very unhealthy	13.00 (2.75%)	8.00 (5.23%)	2.00 (1.32%)	3.00 (1.80%)	<0.001*
Unhealthy	65.00 (13.77%)	38.00 (24.84%)	20.00 (13.16%)	7.00 (4.19%)	
Middle	211.00 (44.70%)	75.00 (49.02%)	78.00 (51.32%)	58.00 (34.73%)	
Healthy	159.00 (33.69%)	29.00 (18.95%)	45.00 (29.61%)	85.00 (50.90%)	
Very healthy	18.00 (3.81%)	2.00 (1.31%)	4.00 (2.63%)	12.00 (7.19%)	
Physical activity (increased heart beats)					
Never	144.00 (30.51%)	61.00 (39.87%)	46.00 (30.26%)	37.00 (22.16%)	<0.001*
Rare	135.00 (28.60%)	44.00 (28.76%)	51.00 (33.55%)	40.00 (23.95%)	
Sometimes	116.00 (24.58%)	33.00 (21.57%)	35.00 (23.03%)	48.00 (28.74%)	
Often	65.00 (13.77%)	15.00 (9.80%)	17.00 (11.18%)	33.00 (19.76%)	
Always	12.00 (2.54%)	0.00 (0.00%)	3.00 (1.97%)	9.00 (5.39%)	

Bold value means a significant *p*-value.

For the total population (*N* = 471), significant differences were observed across habit levels in all constructs, with the high habit group consistently scoring the highest. In terms of attitude, the high habit group had the highest mean score (4.46 ± 0.48), significantly higher than the low habit group (3.84 ± 0.69), with a *p*-value <0.001. Similarly, the high habit group exhibited the highest scores for subjective norm (3.88 ± 0.85), perceived behavioral control (4.16 ± 0.66), and intention (4.17 ± 0.64), all showing statistically significant differences when compared to the low and medium habit groups. For behavior, measured by the number of servings consumed daily, the average of total population is 3.27 ± 0.93, the high habit group reported the highest average (3.73 ± 0.93), while the low habit group consumed fewer servings (2.81 ± 0.83), with a p-value <0.001. The frequency of consumption of 5 serving a day followed a similar trend, with the high habit group consuming fruits and vegetables most frequently (3.25 ± 1.22), while the low habit group had the lowest frequency (1.63 ± 0.78), with all differences being statistically significant. The average of total population is 2.42 ± 1.23 (Table 3).

**Table 3:** Constructs Cronbach’s alpha, mean scores, and standard deviations.

Studied parameters	Alpha	Total *N* = 471	Low habit *N* = 153	Medium habit *N* = 151	High habit *N* = 167	*p*-value
Attitude	0.77	4.14 ± 0.66	3.84 ± 0.69	4.08 ± 0.64	4.46 ± 0.48	<0.001*
Subjective norm	0.83	3.38 ± 0.98	2.93 ± 1.00	3.28 ± 0.86	3.88 ± 0.85	<0.001*
Perceived behavioral control	0.81	3.62 ± 0.90	3.03 ± 0.92	3.61 ± 0.71	4.16 ± 0.66	<0.001*
Intention	0.92	3.45 ± 1.00	2.69 ± 0.98	3.43 ± 0.75	4.17 ± 0.64	<0.001*
Behavior	0.72					
Number of servings daily last week		3.27 ± 0.93	2.81 ± 0.83	3.23 ± 0.76	3.73 ± 0.93	<0.001*
Frequency of consumption		2.42 ± 1.23	1.63 ± 0.78	2.31 ± 1.01	3.25 ± 1.22	<0.001*

Bold value means a significant *p*-value.

### 4.2 Predicting fruits and vegetables consumption with TPB

The hypothesized TPB model fits the data very well (*χ*^2^ (78) = 2597.468, CFI = 0.969, TLI = 0.958, RMSEA = 0.054, SRMR = 0.054) (Table 4). For behavior predictors, the overall model explained 40% of the variance in behavior for the total sample (*R*^2^ = 0.40). Significant predictors of behavior include intention (*b* = 0.24, *p* = 0.012) and perceived behavioral control (*b* = 0.42, *p* < 0.001). For intention predictors, the overall model explained 74% of the variance in intention for the total sample (*R*^2^ = 0.74). The significant predictors included attitude (*b* = 0.15, *p* = 0.025), subjective norm (*b* = 0.19, *p* = 0.001), and perceived behavioral control (*b* = 0.66, *p* < 0.001).

**Table 4:** Results of structural equation models.

Studied parameters	Total *N* = 471				Low habit *N* = 153				Medium habit *N* = 151				High habit *N* = 167			
	*R*^2^	*r*	*b*	*p*	*R*^2^	*r*	*b*	*p*	*R*^2^	*r*	*b*	*p*	*R*^2^	*r*	*b*	*p*
Behavior predictors:	0.40				0.32				0.17				0.09			
Intention		0.52*	0.24	**0.012**		0.22*	0.02	0.921		0.27*	0.04	0.859		0.36*	0.22	0.220
Perceived Behavioral Control		0.25*	0.42	**0.000**		0.15	0.55	**0.002**		0.13	0.38	0.129		0.06	0.10	0.626
Intention predictors:	0.74				0.63				0.62				0.63			
Attitude		0.65*	0.15	**0.025**		0.45*	0.20	0.122		0.48*	0.12	0.313		0.56*	0.14	0.302
Subjective Norm		0.51*	0.19	**0.001**		0.48*	0.12	0.210		0.31*	0.15	0.205		0.37*	0.28	**0.034**
Perceived Behavioral Control		0.36*	0.66	**0.000**		0.35*	0.62	**0.001**		0.24*	0.68	**0.001**		0.18*	0.55	**0.001**

*R*^2^: coefficient of determination, *r*: correlations, *b*: standardized regression coefficients and *p*: *p*-values, *significant correlation at a *p* < 0.05. Bold value means a significant *p*-value.

### 4.3 The role of habit as a moderator of fruits and vegetables consumption

Initial testing of the hypothesized Theory of Planned Behavior (TPB) model demonstrated a good fit for the low habit group [*χ*^2^ (57) = 84.292, CFI = 0.957, TLI = 0.942, RMSEA = 0.056, SRMR = 0.058] and medium habit group [*χ*^2^ (57) = 92.796, CFI = 0.938, TLI = 0.916, RMSEA = 0.064, SRMR = 0.058]. A very good fit was observed for the high habit group [*χ*^2^ (57) = 67.198, CFI = 0.984, TLI = 0.978, RMSEA = 0.033, SRMR = 0.042]. The configural model (baseline model) provided strong overall fit indices [*χ*^2^ (171) = 242.013, CFI = 0.962, TLI = 0.947, RMSEA = 0.051, SRMR = 0.048], supporting the decision to divide the sample into three habit-based subgroups. Measurement invariance testing revealed a slight change in overall model fit for the metric invariance model [Δ*χ*^2^ (16) = 30.27, *p* = 0.017; ΔCFI = 0.003; ΔTLI = 0.005; ΔRMSEA = 0.003, ΔSRMR = 0.015], and a more substantial change for the scalar invariance model [Δ*χ*^2^ (16) = 51.038, *p* < 0.001; ΔCFI = 0.015; ΔTLI = 0.013; ΔRMSEA = 0.007, ΔSRMR = 0.005]. While the difference in *χ*^2^ between the metric and configural models was statistically significant (*p* < 0.05), other fit indices fell within acceptable thresholds. Similarly, the *χ*^2^ difference between the metric and scalar models was significant (*p* < 0.05), but the changes in CFI and TLI did not meet the recommended criteria. Further investigation identified items that operated differently across the habit groups. Once constraints on these items were relaxed, no significant difference was found between the configural and constrained models, allowing the item parameters to be freely estimated in the subsequent nested model. Testing for invariance in the causal structure revealed a significant decline in model fit [Δ*χ*^2^ (46) = 176.71, p < 0.001; ΔCFI = 0.084; ΔTLI = 0.074; ΔRMSEA = 0.033, ΔSRMR = 0.04]. These results suggest that the structural model varies across habit groups, confirming that habit moderates the relationship between intention and behavior (Table 4).

The behavior predictors showed weaker relationships in the low (*R*^2^ = 0.32), medium (*R*^2^ = 0.17), and high (*R*^2^ = 0.09) habit groups, with PBC remaining significant in the low habit group (*b* = 0.55, *p* = 0.002) but not in the medium or high habit groups. In terms of intention predictors, for the low habit group (*R*^2^ = 0.63), attitude (*b* = 0.20, *p* = 0.122) was less predictive, while subjective norm (*b* = 0.12, *p* = 0.210) and perceived behavioral control (*b* = 0.62, *p* = 0.001) remained significant. In the medium habit group (*R*^2^ = 0.62), subjective norm and perceived behavioral control were also significant predictors, whereas attitude was weaker (*b* = 0.12, *p* = 0.313). For the high habit group (*R*^2^ = 0.63), subjective norm (*b* = 0.28, *p* = 0.034) and perceived behavioral control (*b* = 0.55, *p* = 0.001) significantly predicted intention.

Taken together, these results suggest that intention and perceived behavioral control are strong predictors of behavior across all groups, with a stronger effect of PBC observed in the low habit group. The intention predictors, particularly attitude, subjective norm, and perceived behavioral control, were all significant across groups, with subjective norm emerging as a particularly strong predictor in the high habit group and PBC as a strong predictor for the three different groups (Table 4).

### 4.4 Underlying beliefs

For the total population (*N* = 471), behavioral beliefs (e.g., “More energy,” “Higher control over weight”) show significant positive correlations with both attitude and intention, with correlations ranging from 0.31 to 0.58. The highest correlation is observed with the belief that eating fruits and vegetables leads to feeling better (0.57 with attitude), emphasizing its strong influence on attitudes towards consumption. However, this belief has no significant correlation with intention for both medium habit and high habit groups. Normative beliefs (e.g., influence of family and friends) show a consistent positive correlation with both subjective norm (SN) and intention across all habit groups, ranging from 0.45 to 0.61.

**Figure 1:**
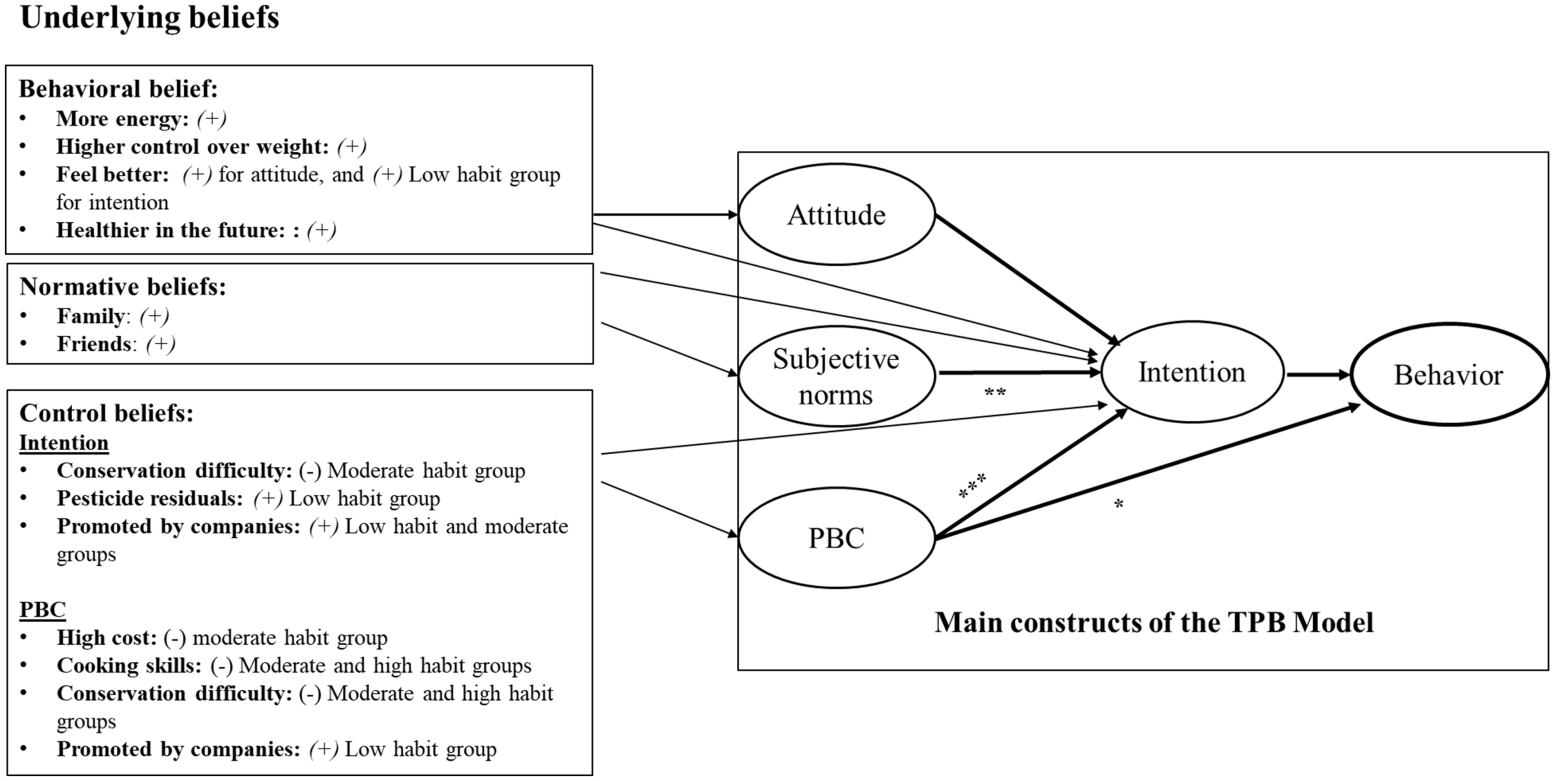
Schematic representation of the main findings. *Significant prediction for low habit group, **significant prediction for high habit group, ***significant for the three groups (low, moderate and high habit groups). (+): positive correlation, (−): negative correlation, PCB, Perceived behavioral control.

Table 5 shows the correlations between control beliefs and PBC and intention related to fruit and vegetable consumption across low, medium, and high habit groups. PBC is negatively influenced by high cost, cooking skills, and conservation difficulty, for overall study population and especially in medium and high habit groups (except for high cost), indicating these factors are perceived as barriers to control. Pesticide concerns do not significantly affect PBC. Beliefs about company promotions positively impact PBC, particularly in the total population and medium habit group. Intention is weakly influenced by high cost, cooking skills, and conservation difficulty, with stronger negative correlations observed in the medium habit group. Concerns about pesticide residues slightly increase intention in the medium habit group. Company promotions have a positive impact on intention, for overall groups and especially in the low and medium habit groups. Main findings are summarized in Figure 1.

**Table 5:** Correlations of beliefs with their relative direct measure.

Studied parameters	Total *N* = 471		Low habit *N* = 153		Medium habit *N* = 151		High habit *N* = 167	
Behavioral beliefs	Attitude	Intention	Attitude	Intention	Attitude	Intention	Attitude	Intention
More energy	0.51*	0.31*	0.58*	0.36*	0.49*	0.17*	0.35*	0.20*
Higher control over weight	0.51*	0.25*	0.55*	0.23*	0.54*	0.21*	0.39*	0.21*
Feel better	0.57*	0.21*	0.54*	0.18*	0.65*	0.09	0.44*	0.15
Healthier in the future	0.58*	0.24*	0.63*	0.25*	0.63*	0.18*	0.49*	0.24*

Studied parameters	Total *N* = 471		Low habit *N* = 153		Medium habit *N* = 151		High habit *N* = 167	
Normative beliefs	SN	Intention	SN	Intention	SN	Intention	SN	Intention
My family	0.59*	0.54*	0.56*	0.46*	0.45*	0.35*	0.54*	0.55*
Friends	0.61*	0.57*	0.59*	0.51*	0.45*	0.40*	0.55*	0.57*

Studied parameters	Total *N* = 471		Low habit *N* = 153		Medium habit *N* = 151		High habit *N* = 167	
Control beliefs	PBC	Intention	PBC	Intention	PBC	Intention	PBC	Intention
High cost	−0.19*	−0.07	−0.15	0.03	−0.34*	−0.15	−0.13	−0.04
Cooking skills	−0.18*	−0.06	−0.02	0.13	−0.25*	−0.10	−0.28*	−0.11
Conservation difficulty	−0.21*	−0.15*	−0.04	0.04	−0.30*	−0.26*	−0.25*	−0.15
Pesticide residuals	−0.04	0.06	−0.09	0.17*	−0.10	−0.05	−0.06	−0.04
Promoted by companies	0.24*	0.32*	0.11	0.31*	0.24*	0.21*	0.03	0.12

*p < 0.05. SN; subjective norm, PBC; perceived behavioral control.

## 5 Discussion

The aim of this study is to provide a comprehensive analysis of fruit and vegetable consumption among Saudi adults using the Theory of Planned Behavior (TPB). Our findings confirm the previously reported low levels of fruit and vegetable consumption among Saudi adults (11, 14) and further emphasize that both the average daily servings and the frequency of meeting the recommended five servings per day are notably low. Additionally, our results suggest that higher fruit and vegetable consumption habits are linked to better knowledge, healthier dietary practices, and more consistent physical activity. In contrast, individuals with low consumption habits tend to show less engagement in these health-promoting behaviors. Consistent with these findings, Le Turc et al. (33) reported that individuals with a deeper understanding of fruits and vegetables demonstrate better dietary habits, supporting the notion that knowledge influences food choices and consumption patterns. Similarly, studies have shown that increased physical activity is associated with higher fruit and vegetable consumption as part of an integrated healthy lifestyle (34, 35).

The hypotheses derived from the Theory of Planned Behavior (TPB) suggested that attitude, subjective norms, and perceived behavioral control (PBC) would significantly predict the intention to consume vegetables. Our study confirmed these predictions, aligning with previous research (19, 36, 37). However, attitude was found to be a less consistent predictor of intention, particularly when the groups were analyzed separately. In contrast, subjective norms emerged as a notably strong predictor in the high-habit group, emphasizing its role as a key factor influencing intentions. PBC, on the other hand, was the only significant predictor across all three habit groups. Furthermore, as hypothesized, both intention and PBC significantly predicted the reported vegetable consumption, as described by Menozzi et al. (37). The hypothesis was confirmed by our findings across all groups, with PBC being particularly influential in the low-habit group. These results highlight the relevance of the TPB in understanding the factors that drive fruits and vegetables consumption, both across different habit levels and within the entire studied population. Taken together, interventions to increase fruits and vegetable eating among adults Saudis can target the three variables attitudes, subjective norms and PBC, since they all significantly correlate with intention, this latter correlates with the studied behavior (eating 5 portions of fruits and vegetables/day).

The TPB suggests that an individual’s beliefs about the expected outcomes of an action (behavioral beliefs), the expectations of others (normative beliefs), and the factors that could either facilitate or hinder the execution of the action (control beliefs) influence their attitudes, subjective norms, and perceived behavioral control (PBC). These factors then play a role in shaping intentions and behavior (38). Investigating the relationship between these key beliefs and their corresponding measures, as well as their connection to intentions, offers valuable insights into how to design effective interventions. Ideally, the intervention should focus on the beliefs that have the greatest impact on predicting intentions. In our study, behavioral beliefs (such as the benefits of increased energy or weight control, healthier in the future) are significantly correlated with both attitudes and intentions over all the sample. However, this belief does not significantly correlate with intention in individuals with medium or high habits. On the other hand, normative beliefs (such as the influence of family and friends) show a consistent positive correlation with both subjective norms and intentions across all habit groups. Regarding control beliefs, our findings indicate that they primarily influence PBC rather than intention, with distinct patterns observed across the three habit groups. External factors, such as promotions, were found to influence PBC, particularly among individuals with lower and medium consumption habits. In contrast, internal factors, such as cooking skills and conservation difficulty, had a more significant impact on PBC in the low and high consumption habit groups. These results underscore the importance of considering different types of beliefs—both external and internal—in shaping attitudes and intentions, offering valuable insights for developing targeted strategies to promote behavior change.

The data strongly supports the idea that habit plays a foundational role in shaping all aspects of the TPB when it comes to fruit and vegetable consumption. Regular consumers (high habit group) show more positive attitudes, stronger subjective norms, higher perceived behavioral control, and higher intentions to consume fruits and vegetables. These factors all contribute to their actual higher consumption levels. On the other hand, individuals with low habits are less influenced by these psychological constructs, making it harder for them to increase their intake. In fact, Labadorf (39) described the use of habit as an added explanatory power to the TPB model. Hence, from a practical perspective, interventions targeting low habit consumers may need to focus on boosting their confidence (PBC), shaping positive attitudes, and creating supportive social norms to help them develop the intention and actual behavior of consuming more fruits and vegetables. Establishing small, manageable habits could gradually lead to changes in these constructs, ultimately fostering healthier consumption patterns.

The current study has some limitations that should be acknowledged. First, the use of cross-sectional data and self-reported measures introduces potential concerns regarding the causal relationships within the TPB framework, which may have led to an overestimation of the associations between TPB variables and behavior. However, this approach is commonly employed in TPB research (19), and the validity and reliability of the questionnaire were tested, suggesting that the data collected is reasonably accurate. In addition, our TPB model provided good predictions regarding the intention to consume five servings of fruits and vegetables daily among young adults, explaining 74% of the variance. It also effectively predicted self-reported fruit and vegetable consumption behavior, accounting for 40% of the variance. The high quality of the psychometric tools used to assess psychosocial and behavioral factors may have contributed to the accuracy of these predictions. A longitudinal design could help determine whether changes in perceived behavioral control would lead to increased fruit and vegetable intake over time, thus strengthening causal claims. As for the reliance on self-reported data, the potential bias could be mitigated by incorporating objective measures (e.g., food diaries, biomarkers) in future studies to complement self-reported data and reduce reporting bias. A second potential limitation is the convenience sampling technique used in the study may affect the generalizability of the findings (40). This limitation is further compounded by the use of social media platforms (e.g., WhatsApp, Telegram), which might bias the sample toward younger or more tech-savvy individuals. Despite these limitations, the study provides valuable insights into the factors influencing fruit and vegetable consumption in Saudi Arabia, particularly in the context of declining intake. Additionally, it offers important guidance for policymakers and companies in designing targeted interventions for individuals with low fruit and vegetable consumption habits. Future studies might consider employing probability sampling to enhance the generalizability of the findings.

## 6 Conclusion

This study examined the applicability of the TPB model in predicting the consumption of the recommended five servings of fruits and vegetables per day among adults in Saudi Arabia, as advised by the Ministry of Health. It also identified key factors influencing this behavior and suggested potential intervention strategies. On average, both the number of servings and the frequency of meeting the five servings per day recommendation were found to be low. The study confirmed that habits play a crucial role in shaping the various constructs of the TPB. PBC emerged as a significant predictor of fruit and vegetable consumption behavior among individuals with low habits. For all groups, PBC was identified as the primary determinant of intention, with social norms also playing a key role for the high-habit group. This indicates that fruit and vegetable consumption is driven by both intention and PBC, with individual habits being an important consideration.

To increase fruits and vegetables across all the groups, enhancing the PCB is key and intervention should be tailored. For low-habit consumers, interventions should focus on strengthening control beliefs related to external factors. Moreover, barriers such as cost, lack of cooking skills, and preservation challenges should be addressed by providing affordable options, simple cooking tips, and strategies for better preservation, particularly for low-habit individuals. Finally, interventions should encourage gradual changes and provide practical tools (e.g., meal plans, preparation tips) to facilitate the transition from low to medium and high habit groups.

For individuals in the high-habit group, promoting personal skills, such as preservation techniques, may help sustain high consumption levels. Additionally, enhancing knowledge and fostering an overall healthy lifestyle could be effective strategies in helping the Saudi population meet the recommended five servings of fruits and vegetables per day. Interventions should also aim to strengthen family and friend support, particularly for individuals in the high-habit group, where these social influences are most impactful. This is important, as fruit and vegetable intake is generally low among the entire population.

For further research, it is recommended to expand the analysis by incorporating external factors such as socio-economic status, cultural influences, and the availability of fruits and vegetables. This approach would provide a more comprehensive understanding of the determinants of this dietary behavior.

## Data Availability

The raw data supporting the conclusions of this article will be made available by the authors, under reasonable request.
